# All-cause mortality in patients with medullary thyroid carcinoma of different ages: an inverse L-curve analysis study

**DOI:** 10.3389/fendo.2025.1574985

**Published:** 2025-06-10

**Authors:** Jiahua Chen, Jiafei Chen, Mi Zhang, Yong Hong

**Affiliations:** ^1^ Department of Thyroid and Breast Surgery, Nanxishan Hospital of Guangxi Zhuang Autonomous Region, Guilin, China; ^2^ Department of General Medicine, Shazi Health Center, Liuzhou, Guangxi Zhuang Autonomous Region, China

**Keywords:** medullary thyroid cancer, age, SEER database, nonlinear relationship, all due to death

## Abstract

**Background:**

Medullary thyroid carcinoma (MTC) is a malignancy with a high mortality rate and a wide age range. However, there are relatively few studies on the relationship between age and all-cause mortality in patients with MTC. As one of the important factors influencing cancer prognosis, the association between age and all-cause mortality in MTC patients needs to be further investigated.

**Objective:**

The aim of this study was to investigate the relationship between age and all-cause mortality in MTC patients, especially whether there is an inverse L-shaped curve relationship, in order to provide new insights for clinical management and prognostic assessment.

**Methods:**

A detailed retrospective cohort analysis of 1291 MTC patients diagnosed between 2000 and 2021 was included in this study using the Surveillance, Epidemiology, and End Results (SEER) database. Cox regression modelling, curve fitting, Kaplan-Meier (KM) survival curves and subgroup analyses were used to assess the association between age and all-cause mortality in MTC patients. Potential confounders, including patient sex, race, Summary stage, surgery, Lymph.node.dissection, tumour size and lymph node metastasis (LNM), were rigorously controlled.

**Results:**

The risk of all-cause mortality in MTC patients increased by 6% per 1-year increase in age (hazard ratio HR=1.06, 95% confidence interval CI: 1.05-1.06, p<0.001). Further analysis revealed a significant inverse L-shaped relationship between age and all-cause mortality in MTC patients. Specifically, before the age of 50 years, the hazard ratio increased slowly with age (HR=1.024, 95% CI: 0.991-1.059) and the difference was not statistically significant (p=0.1616). After the age of 50 years, the hazard ratio accelerated with increasing age (HR=1.066, 95% CI: 1.051-1.081) and the difference was statistically significant (p<0.001).

**Conclusion:**

The results of this study confirm that there is an inverse L-shaped relationship between age and all-cause mortality in MTC patients. The risk of all-cause mortality in MTC patients increased significantly with age after age >50 years. This finding provides new insights into understanding the complex relationship between age and all-cause mortality in MTC, which may help inform clinical management and prognostic assessment.

## 1 Introduction

Medullary thyroid carcinoma (MTC) is a rare neuroendocrine tumour that originates from the parafollicular (C) cells of the thyroid gland (1). It accounts for approximately 3% of all thyroid cancers (2–4). Its clinical presentation differs significantly from the more common differentiated thyroid cancer. Based on aetiology, MTC can be divided into sporadic MTC (sMTC, approximately 75%) and hereditary MTC (hMTC, approximately 25%) (5–7). Among these, hereditary MTC is associated with several inherited diseases, such as multiple endocrine neoplasia type 2 (MEN2) (8, 9). Due to its high aggressiveness, MTC is prone to early lymphatic metastasis and extensive systemic spread, leading to a poor prognosis (10). In fact, MTC accounts for approximately 13% of thyroid cancer deaths (10–14), a proportion that underscores the urgency of advancing research into MTC. The age of onset of MTC patients spans a wide age range, and age has been shown to be a key factor influencing the progression and prognosis of MTC (15). However, due to the low proportion of MTC among thyroid cancers, there is a paucity of relevant data, which limits the in-depth understanding of the age-related prognosis of MTC patients as well as the optimisation of clinical management. Therefore, there is a strong need for large sample studies to fill this gap. The aim of this study was to investigate whether all-cause mortality in MTC patients is associated with age and related confounders, and to further analyse whether there is a non-linear relationship between age and all-cause mortality in MTC patients. To this end, we conducted a retrospective cohort study of 1291 cases of medullary thyroid carcinoma using the Surveillance, Epidemiology, and End Results (SEER) database.

### 1.1 Data extraction

In our study, we chose age as the main relevant exposure variable. The aim of our study was to examine the relationship between age and all-cause mortality in patients with cancer. Potential confounders include: sex, race, summary stage, surgery, lymph node dissection,tumour size and lymph node metastasis. Data were extracted using SEER stat version 8.4.4. Data extracted from SEER Research Data, 17 registries, Nov 2023 Sub (2000-2021). 4066 patients with medullary thyroid carcinoma with ICD-0-3/WHO 2008 as thyroid and histology of 8510/3 and 8345/3 were included. Excluded:1. Autopsy and death certificate only 2. Not one primary only3.Age<20 4. Race missing 5. Summary stage missing 6. Primary site surgery missing 7. Number of lymph node dissection missing 8. Tumour size missing 9. Tumour size>200mm, tumour size<1mm 10. Lymph node missing:1291 eligible patients were included in the SEER primary cohort. All codes from the SEER database at https://seer.cancer.gov/.

### 1.2 Statistical analysis

All statistical analyses were performed using Free Statistics software (version 2.0). Categorical variables were expressed as frequencies and corresponding percentages, and continuous variables were expressed as means and standard deviations (SDs). After covariate selection, Cox proportional risk regression models were used to assess overall survival (OS) and cancer-specific survival (CSS) of medullary thyroid cancer patients at different ages. In this context, OS is defined as the time from diagnosis to death from any cause; and CSS refers specifically to the time from diagnosis to definitive death from medullary thyroid carcinoma (MTC). In this study, age was primarily used as a continuous variable in the analyses. Survival status was coded as a dichotomous variable, i.e. alive or dead. In multivariate Cox regression analyses, we adjusted for potential confounders, including sex, race, summary stage, surgery, lymph node dissection, tumour size, and lymph node metastasis. The results of the analyses were presented as hazard ratios (HRs) and their 95% confidence intervals (CIs). In addition, we further explored how the variables of gender and ethnicity affected age-related survival outcomes through subgroup analyses. In this study, the statistical significance level was set at p < 0.05.

## 2 Results

### 2.1 Patient characteristics

**Figure 1:**
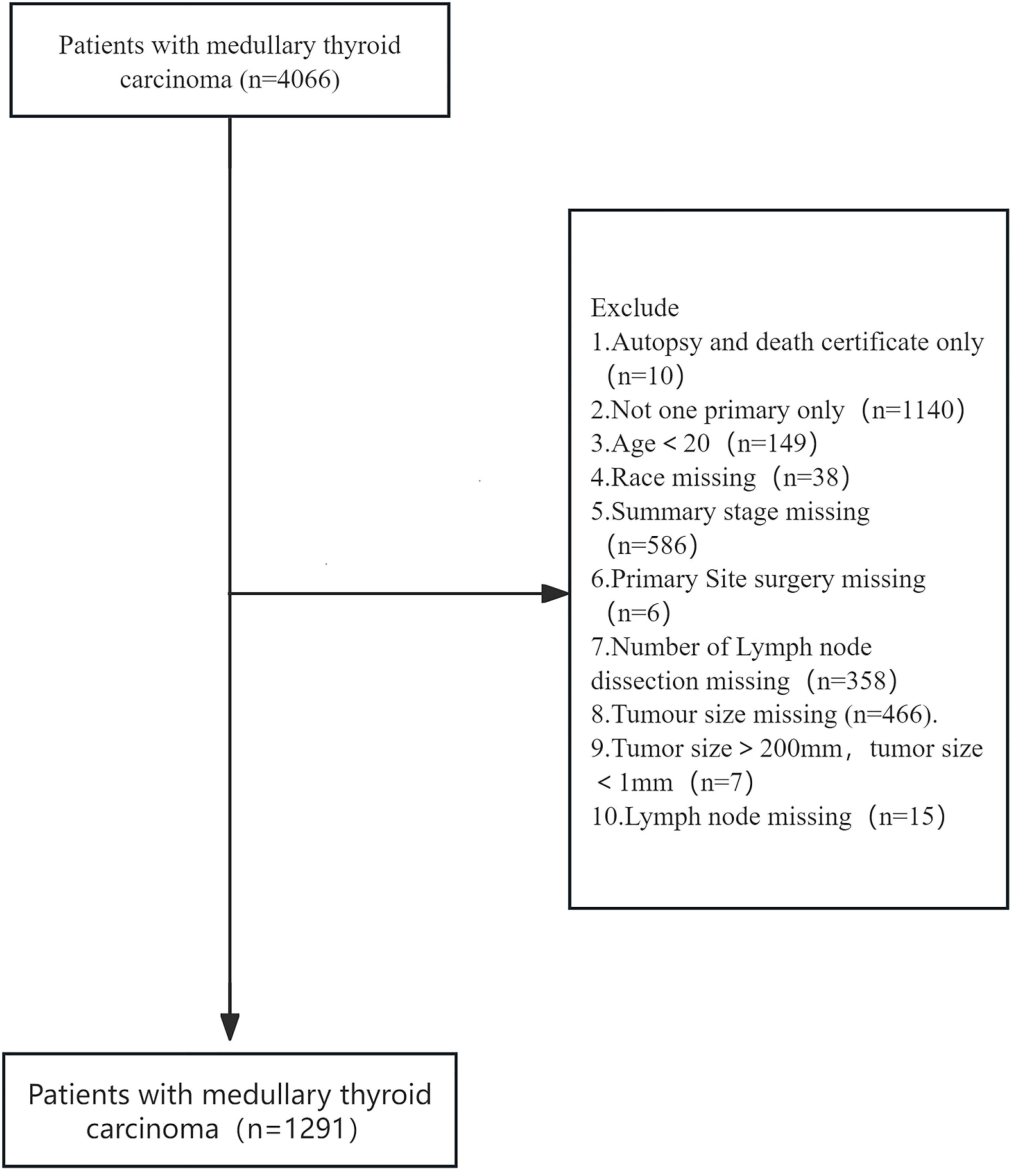
Flow diagram of inclusion and exclusion.

Based on established inclusion criteria, we screened patient records for medullary thyroid cancer (MTC) from the SEER database (2000-2021) for ICD-O-3 codes 8510/3 and 8345/3. Inclusion criteria included a confirmed diagnosis of MTC and the availability of complete clinicopathological information. In the end, we constructed a cohort of 1291 patients. **Figure 1** shows the detailed patient selection process, while **Table 1** summarises the demographic characteristics of each patient group. In this study cohort, the mean age of the 1291 patients was 51.0 ± 15.2 years, of which 301 patients died, resulting in a mortality rate of 23.32%. The mean age of the deceased patients was 60.0 ± 15.2 years, which was significantly higher than the mean age of the surviving patients (48.2 ± 14.1 years, p < 0.001). This suggests that there may be a significant association between age and risk of death in MTC patients. Further analysis revealed significant differences between patients in the death and survival groups on several variables, including age, sex, tumour stage, surgery, lymph node metastasis and tumour size. These differences suggest that these variables may be potential factors influencing the prognosis of MTC patients.

**Table 1:** Baseline characteristics of participants.

Variables	Total (n = 1291)	Alive (n = 990)	death (n = 301)	p	statistic
Age, Mean ± SD	51.0 ± 15.2	48.2 ± 14.1	60.0 ± 15.2	< 0.001	153.181
Sex, n (%)				< 0.001	36.852
Female	785 (60.8)	647 (65.4)	138 (45.8)		
Male	506 (39.2)	343 (34.6)	163 (54.2)		
Race, n (%)				0.087	2.934
Other	199 (15.4)	162 (16.4)	37 (12.3)		
White	1092 (84.6)	828 (83.6)	264 (87.7)		
Summary stage, n (%)				< 0.001	241.483
Distant	150 (11.6)	46 (4.6)	104 (34.6)		
Localized	702 (54.4)	625 (63.1)	77 (25.6)		
Regional	439 (34.0)	319 (32.2)	120 (39.9)		
Surgery, n (%)				< 0.001	99.968
No	56 (4.3)	12 (1.2)	44 (14.6)		
Yes	1235 (95.7)	978 (98.8)	257 (85.4)		
Lymph.node.dissection, n (%)				0.452	1.589
1 to 3 regional lymph nodes removed	178 (13.8)	142 (14.3)	36 (12)		
4 or more regional lymph nodes removed	775 (60.0)	595 (60.1)	180 (59.8)		
None	338 (26.2)	253 (25.6)	85 (28.2)		
Lymph node metastasis, n (%)				< 0.001	88.731
No	737 (57.1)	636 (64.2)	101 (33.6)		
Yes	554 (42.9)	354 (35.8)	200 (66.4)		
Tumor, Median (IQR)	22.0 (12.0, 35.0)	19.0 (10.0, 31.0)	32.0 (18.0, 50.0)	< 0.001	101.337

### 2.2 Core regression results

#### 2.2.1 Univariate analysis of age and all-cause mortality in MTC patients

Univariate Cox regression analysis (see **Table 2**) showed that the risk of all-cause mortality in patients with medullary thyroid cancer (MTC) increased by 5% (risk ratio HR=1.05, 95% confidence interval CI: 1.05-1.06) for every 1-year increase in age (p-value <0.001).

**Table 2:** Univariate analysis of age and all-cause mortality in MTC patients.

Item	HR (95%CI)	P (Wald’s test)	P (LR-test)
Age	1.05 (1.05,1.06)	< 0.001	< 0.001

#### 2.2.2 Multifactorial analysis of age and all-cause mortality in MTC patients

To more fully assess the effect of age on the risk of all-cause mortality in medullary thyroid cancer (MTC), we constructed three different Cox risk models that progressively adjusted for potential confounders as follows: see **Table 3**. Model 1: Only sex and race were included as confounders. cox regression analyses showed that each 1-year increase in age was associated with a 5% increase in all-cause mortality in patients at risk for MTC (risk ratio HR=1.05, 95% confidence interval CI: 1.05-1.06, p-value <0.001). Model 2: After further adjustment for tumour stage (summary stage) and surgical status (surgery) based on model 1, Cox regression analysis (see **Table 3**) showed that the risk of all-cause mortality in patients with MTC increased by 6% with each additional year of age (hazard ratio HR=1.06, 95% confidence interval CI: 1.05-1.07, p-value <0.001). Model 3: Further adjustments for lymph node dissection, tumour size and lymph node metastasis (LNM) were made based on model 2. Cox regression analyses (see **Table 3**) showed that for each 1-year increase in age, the risk of all-cause mortality in MTC patients increased by 6% (hazard ratio HR=1.06, 95% confidence interval CI: 1.05-1.07, p-value <0.001).

**Table 3:** Multifactorial analysis of age and all-cause mortality in MTC patients.

Variable	Model 1		Model 2		Model 3	
	HR (95%CI)	P-value	HR (95%CI)	P-value	HR (95%CI)	P-value
Age	1.05 (1.05~1.06)	<0.001	1.06 (1.05~1.07)	<0.001	1.06 (1.05~1.06)	<0.001

Model 1: Adjusted for sex and race.

Model 2: Model 1 plus adjustments for summary stage, Surgery.

Model 3: Model 2 plus adjustments for lymph node dissection, Tumor size, and LNM.

By progressively adjusting for confounders, we found that the effect of age on the risk of all-cause mortality in MTC remained consistent across models and that the risk estimates became more precise with the progressive inclusion of confounders. This suggests that age is an independent predictor of the risk of all-cause mortality in MTC.

#### 2.2.3 Curve fitting and turning point test

**Figure 2:**
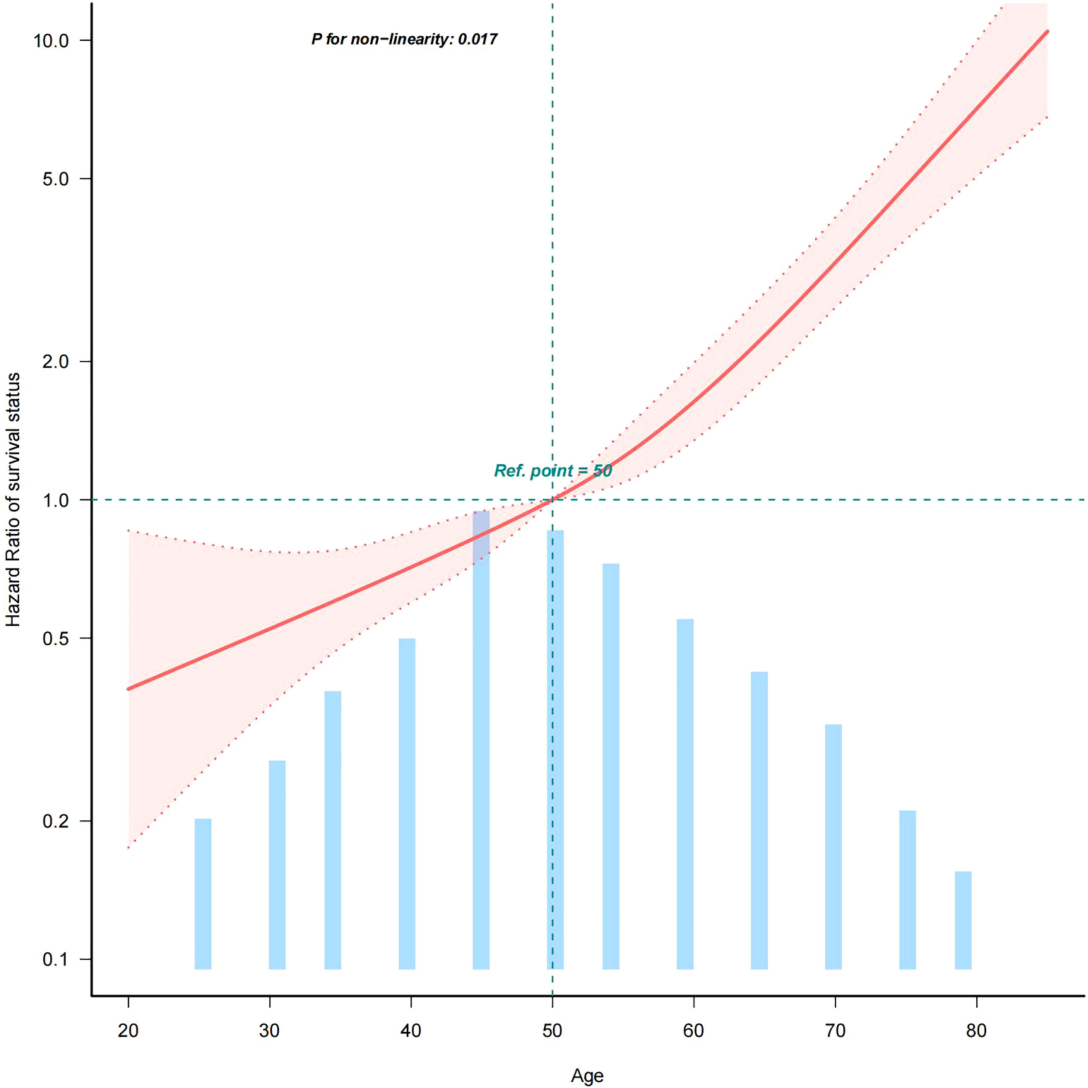
Curve fit of the relationship between age and all-cause mortality in MTC (age quantified with an upper limit of 100%). This figure shows the Hazard Ratio after multivariate adjustment for the association of the continuous variable AGE with all-cause mortality in medullary thyroid cancer.Solid deep red lines are multivariable-adjusted HR. Light red areas are the 95% CI derived from restricted cubic spline regressions with 4 knots.Horizontal light green dotted lines are reference lines for a hazard ratio of 1.0.All-cause mortality from medullary thyroid cancer increases with age. 100% of data shown. Adjusted for sex, race, summary stage, surgery, lymph node dissection, tumour size and LNM.Abbreviations: HR: hazard ratio, CI: confidence interval.

In model 3, we adjusted for confounders such as sex, race, Summary stage, surgery lymph.node dissection, tumour size and lymph node metastasis. Based on the adjusted model 3, we further analysed the association between age and all-cause mortality in medullary thyroid cancer (MTC). The results showed a statistically significant inverse L-shaped relationship between age and MTC all-cause mortality (P < 0.001, see **Figure 2**). Specifically, when age was less than 50 years, MTC all-cause mortality increased slowly with age, with a hazard ratio (HR) of 1.024 (95% confidence interval CI: 0.991-1.059), but the difference was not statistically significant (p = 0.1616). When age was greater than 50 years, all-cause mortality from MTC increased significantly with age, with a hazard ratio (HR) of 1.066 (95% confidence interval CI: 1.051-1.081, p < 0.001, see **Table 4**). This result suggests that before the age of 50 years, the effect of age on all-cause mortality in MTC was small and non-significant, whereas after the age of 50 years, the risk of all-cause mortality in patients with MTC was significantly increased by 6.6% for every 1-year increase in age. This non-linear relationship suggests that in clinical practice, more attention should be paid to the age-related prognostic risk of MTC patients over 50 years of age.

**Table 4:** **Turning point analysis** (age was quantified using the upper limit of 100%).

Turning point	Breakpoint.OR (95%CI)	P value
<50 year	1.024 (0.991,1.059)	0.1616
≥50years	1.066 (1.051,1.081)	< 0.001
Likelihood Ratio test	–	0.046

#### 2.2.4 Survival analysis

**Figure 3:**
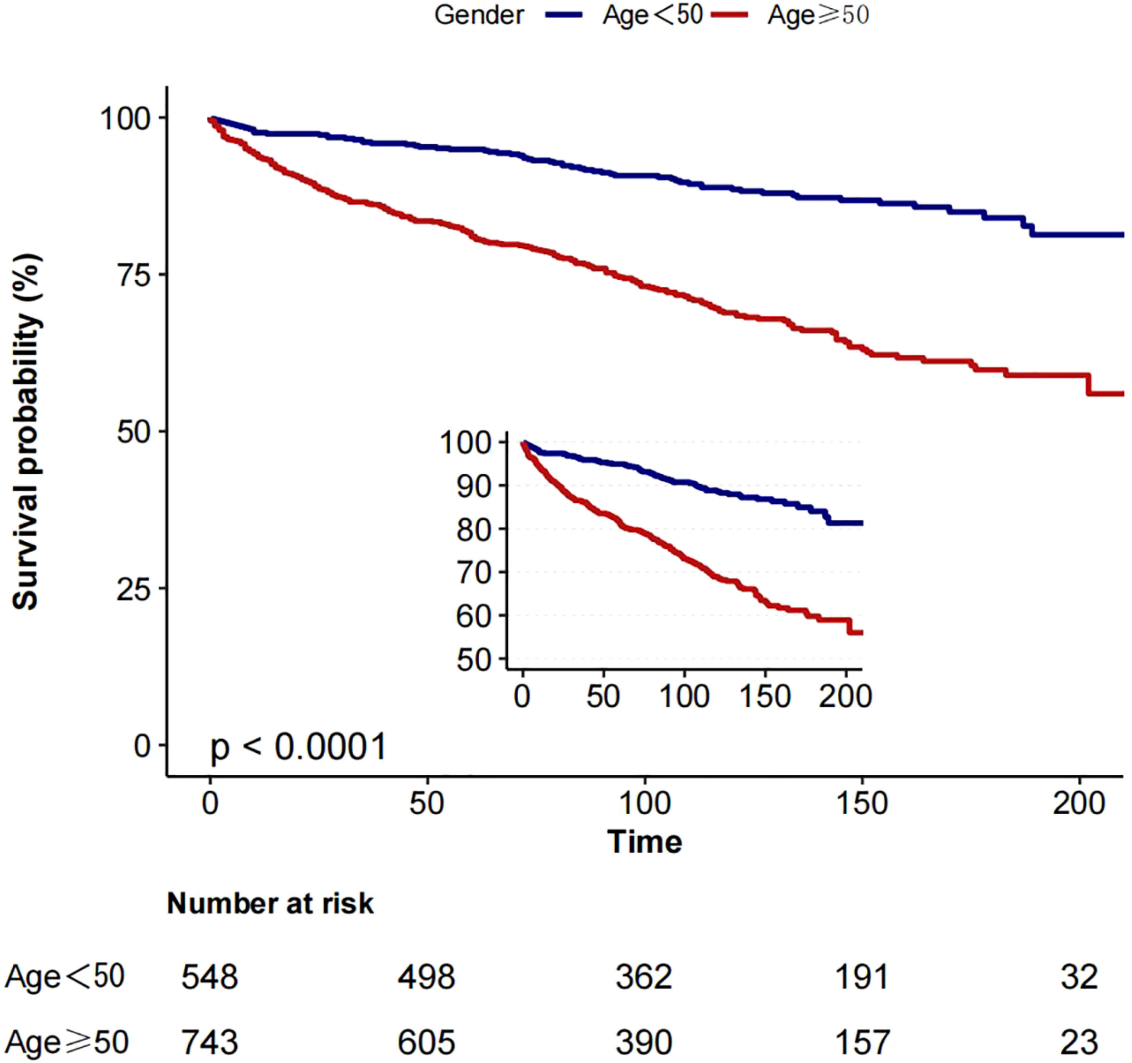
Kaplan-Meier curves: comparison of overall survival for patients in group 1 (age <50) and group 2 (age ≥50).

**Figure 4:**
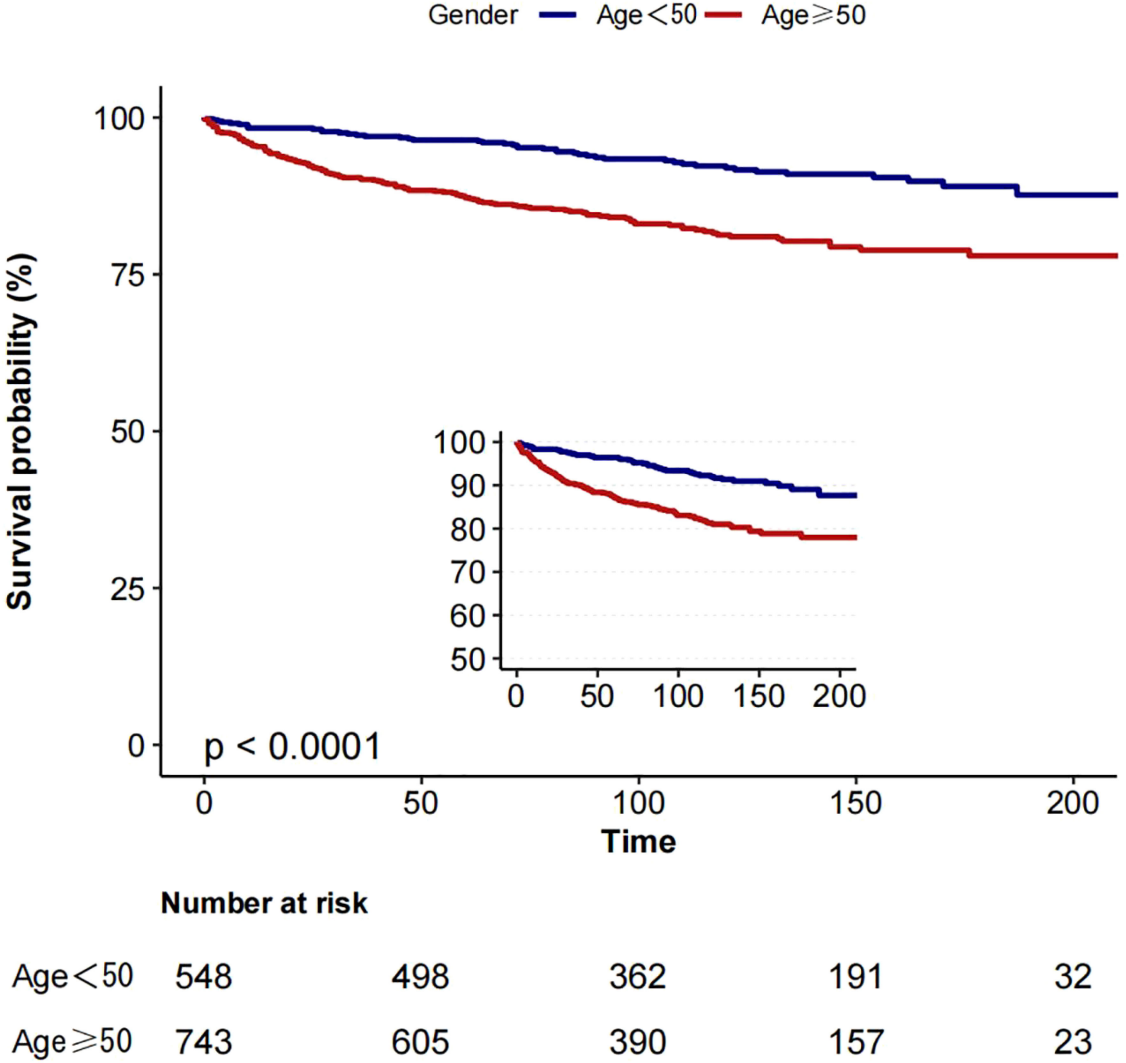
Kaplan-Meier curves: comparison of cancer-specific for patients in group 1 (age <50) and group 2 (age ≥50).

Based on the inflection point of age (50 years), we divided the patients into two groups: group 1 (age <50 years) and group 2 (age ≥50 years). In a cohort of 1291 patients, follow-up was 215 months. The 5-year overall survival (OS) was 94.9% (group 1) and 81.0% (group 2), as shown in **Figure 3**, while the 5-year cancer-specific survival (CSS) was 96.4% (group 1) and 87.2% (group 2) in the two cohorts, as shown in **Figure 4**.

In assessing CSS, we found a competing risk between tumour-related and non-tumour-related deaths. To better reflect this competing relationship, we used a multifactorial competing risk model for our analysis (see **Table 5**). The results showed that the risk of CSS in patients with medullary thyroid cancer (MTC) increased by 3% per 1-year increase in age (risk ratio HR=1.03, 95% confidence interval CI: 1.02-1.04, p-value <0.001). This result further suggests that after taking into account competing risks of non-tumour death, the risk of tumour-specific death in patients with MTC still increases significantly with age and that this increase is statistically significant.

**Table 5:** A multifactorial competing risk model for tumour-specific survival.

Variable	n.total	n.event_%	Followup.Time	crude.HR (95%CI)	crude.P value	adj.HR (95%CI)	adj.P value
Age	1291	174 (13.5)	144023	1.03 (1.02~1.04)	<0.001	1.03 (1.02~1.04)	<0.001

#### 2.2.5 Subgroup analyses

**Figure 5:**
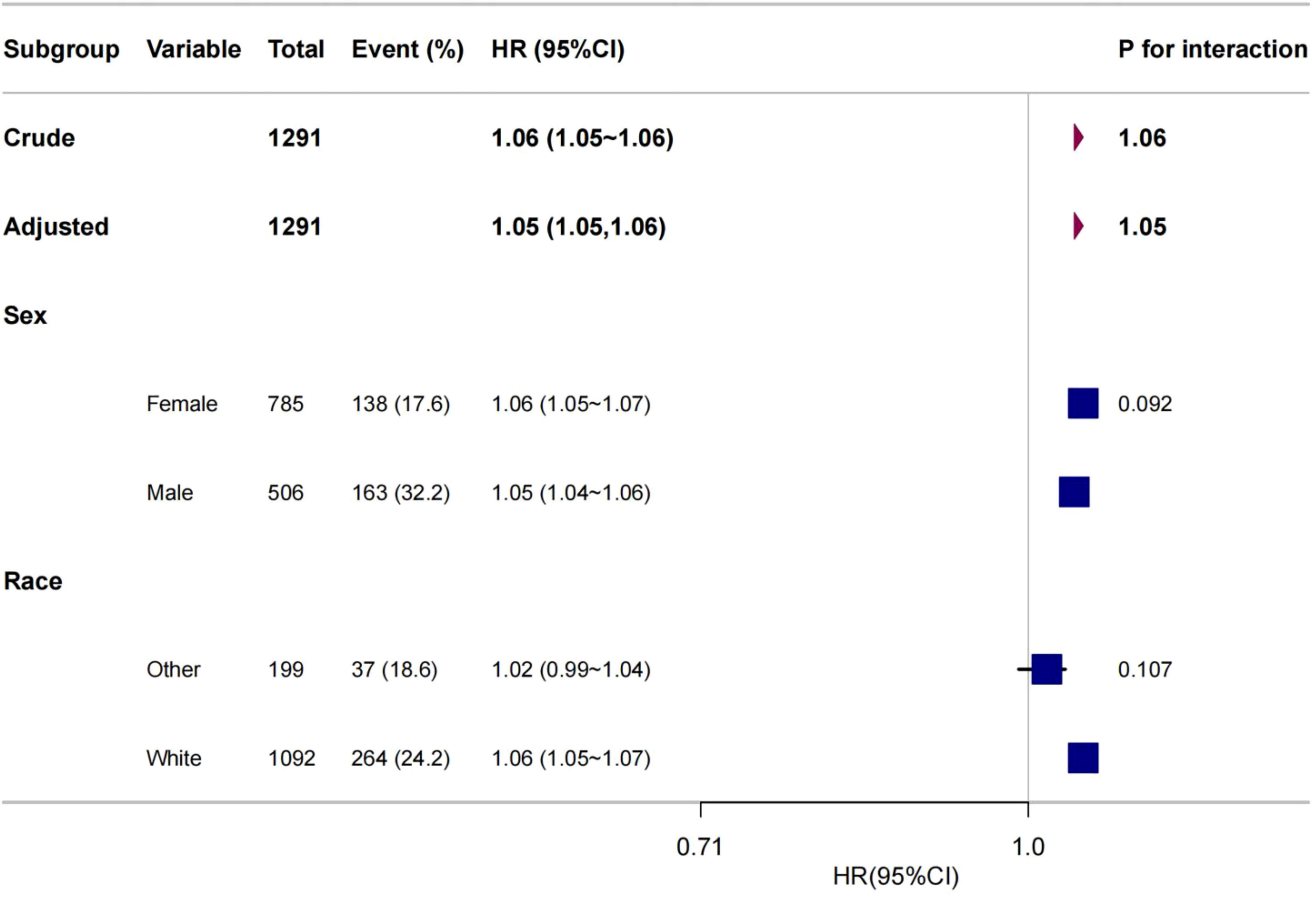
Forest plot of age versus MTC all-cause mortality in different subgroups.

To further explore the association between age and all-cause mortality in medullary thyroid cancer (MTC), this study performed subgroup analyses including variables such as sex and race (see **Figure 5**). We performed 2-test subgroup analyses for sex and ethnicity in our study. To control for the risk of false positives, we adjusted the p-value threshold using the Bonferroni correction method. Dividing the original significance level (0.05) by the number of tests (2) gave an adjusted p-value threshold of 0.025. The results were assessed as follows: the p-value for the gender subgroup was 0.092, which was greater than the adjusted p-value threshold of 0.025, and for the ethnicity subgroup the p-value was 0.107, which was also greater than the adjusted p-value threshold of 0.025. Thus, none of the results were statistically significant at the adjusted p-value threshold. statistically significant. The results of the statistical analyses showed that there were no statistically significant interactions between age and all-cause mortality in the gender and race subgroups. This suggests that the effect of age on all-cause mortality in MTC is consistent across gender and race subgroups, with no significant heterogeneity.

## 3 Discussion

### 3.1 A summary of the findings of the study is presented below

In this study, we explored the complex relationship between age and all-cause mortality in patients with medullary thyroid cancer (MTC) through a detailed retrospective cohort analysis of the SEER database. In this study, we used a curve-fitting technique to reveal an inverse L-shaped curve relationship between these two variables. Specifically, when age was less than 50 years, all-cause mortality in MTC increased slowly with age, but the difference was not statistically significant (p > 0.05). However, when age was greater than 50 years, all-cause mortality in MTC increased significantly with age and the difference was statistically significant (p < 0.001). This finding not only deepens our understanding of the biological characteristics of MTC, but also provides an important basis for the clinical development of personalised treatments based on age inflection points.

### 3.2 This study differs from previous research in a number of ways

In this retrospective cohort study, we systematically examined the relationship between survival outcomes in medullary thyroid cancer (MTC) cases and patient age using the comprehensive Surveillance, Epidemiology, and End Results (SEER) database, a key dataset maintained by the National Cancer Institute. The database provides clinical information on a large sample of patients from 1291 cases, increasing the generalisability and reliability of our conclusions (16–21). Previous studies have documented significant differences in survival between the paediatric and adult populations, highlighting the centrality of age in prognostic decisions (21). Previous studies have amply demonstrated that paediatric cohorts have a significant survival advantage in terms of OS and CSS compared to adult cohorts (22), further emphasising age as an important prognostic determinant in MTC10. In contrast to these pioneering efforts, our study provides unique contributions and insights in multiple dimensions that contribute to a deeper understanding of the topic.

First, previous studies have shown that age is one of the prognostic factors for patients with medullary thyroid carcinoma (MTC). The present study not only confirmed this idea, but also further clarified the quantitative relationship between age and the risk of all-cause mortality in MTC using Cox regression analysis: for every 1-year increase in age, the risk of all-cause mortality in MTC patients increased by 6% (hazard ratio HR = 1.06, 95% confidence interval CI: 1.05-1.07, p < 0.001). In addition, this study found a complex non-linear relationship between age and all-cause mortality in MTC, manifested as an inverted L-shaped curve. This finding is an innovative contribution to the field. Specifically, when age was less than 50 years, all-cause mortality in MTC increased slowly with age, but the difference was not statistically significant (HR = 1.024, 95% CI: 0.991-1.059, p = 0.1616). However, at age >50 years, all-cause mortality from MTC increased significantly with age (HR = 1.066, 95% CI: 1.051-1.081, p < 0.001). This novel perspective not only deepens our understanding of the biological behaviour of MTC, but also provides an important theoretical basis for the development of clinical risk assessment and intervention strategies.

Second, due to the low incidence of medullary thyroid cancer (MTC), there is relatively little clinically available data. This study ensured the reliability and generalisability of the findings by including a large amount of patient data using the Surveillance, Epidemiology, and End Results (SEER) database managed by the National Cancer Institute. This large sample size provides a solid basis for in-depth investigation of the clinical characteristics and prognostic factors of MTC.

### 3.3 Clinical usefulness of this study

The results of this study have important implications for clinical practice, and surveillance strategies can be significantly optimised by accurate risk stratification. For high-risk individuals ≥50 years of age, it is recommended to establish a ‘3+X’ intensive surveillance model, i.e. thyroid ultrasound and serum calcitonin every 3 months, CT scan of the lungs, neck, abdomen and head every 6 months, and whole-body bone scan every year. For low-risk individuals <50 years of age, thyroid ultrasound and serum calcitonin were performed every 6 months, and CT scans of the lungs, neck, abdomen, and head, and whole-body bone scans were performed annually. For patients with no metastatic recurrence after 5 years of follow-up, thyroid ultrasound and serum calcitonin testing can be extended to yearly, ensuring effective monitoring and rational allocation of medical resources.

### 3.4 Changes in the immune microenvironment with age and mechanisms of inverted L-curve

In the field of immune senescence interacting with the tumour microenvironment, recent studies have provided insights into its multi-level biological mechanisms (23, 24). Ageing triggers T-cell immune remodelling, which is strongly associated with poor clinical outcomes in age-related diseases such as cancer. Increased myelopoiesis and lymphoid tissue remodelling are accentuated with age, and the synergistic effect of these two factors creates an immunosuppressive environment that can have a significant negative impact on tumour immunity. At the same time, ageing is associated with thymic degeneration and defective T cell development, which directly limits the diversity of T cells needed to fight tumours. In addition, T cell function declines with age and exhibits a number of senescent features, including metabolic dysfunction, genomic instability, telomere shortening and cellular senescence. In addition, senescent T cells show a lack of adaptability in the immune response process, resulting in varying degrees of decline in their ability to respond to cancer, infection and autoimmune disease (23).

In addition, studies have shown that the senescent microenvironment has a significant impact on tumour progression. Under normal physiological conditions, senescence-related changes can contribute to the transition of tumour cells from an initial or slow-growing state to a highly aggressive and metastatic disease. The accumulation of senescent cells in aged tissues is a key feature of this process. These senescent cells not only undergo extensive epigenetic gene expression changes, but also significantly increase the secretion of pro-inflammatory cytokines, chemokines and growth factors. The senescence-associated secretory phenotype (SASP), which consists of a group of factors secreted by senescent cells, including IL-6, IL-8 and growth factors, has the potential to promote tumour growth and metastasis in the tumour microenvironment. With increasing age, the immune function of the human body gradually declines and the ability of the immune system to recognise and eliminate senescent cells decreases, which in turn promotes the accumulation of senescent cells in tissues (24).

Taking the above mechanisms together, we hypothesise that the particular shape of the inverted L-shaped curve may reflect the following process. In the early stages of ageing (<50 years of age), the diversity of the T cell repertoire maintains basic immunosurveillance functions. However, once the cumulative senescence burden crosses a critical threshold (≥50 years), the SASP-driven immunosuppressive microenvironment and T-cell exhaustion dominate the kinetics of tumour progression. This non-linear transition may be a key intrinsic mechanism for the formation of this curve pattern.

### 3.5 Limitations of the study

Unfortunately, the present study is not without limitations. As a retrospective study based on the SEER database, the present study is inevitably subject to the biases inherent in this type of design, which may result in an uneven representation of the patient cohort and limit the broad applicability of our findings. Despite our efforts to mitigate the effects of confounding variables through multivariate analyses, the lack of data on patients’ body mass index (BMI), complex patterns of comorbidities, and precise details of therapeutic interventions (including the modality and dose of radiotherapy and chemotherapy) in the SEER database may have hindered the full elucidation of the mechanisms underlying the survival differences and may have biased our results. Finally, no sensitivity analyses were performed. Failure to perform sensitivity analyses may lead to increased reliance on statistical modelling, which may introduce a degree of bias. Future research could build on the results of this study and continue with multi-centre studies in European and Asian populations.

### 3.6 Conclusion

In conclusion, this study demonstrated a significant association between age and the risk of all-cause mortality in medullary thyroid cancer (MTC) through a retrospective cohort analysis based on the SEER database. Specifically, the risk of all-cause mortality in MTC patients increased by 6% for every 1-year increase in age (HR=1.06, 95% CI: 1.05-1.06, p<0.001). In addition, the present study further confirmed an inverse L-shaped relationship between age and all-cause mortality in MTC: before the age of 50 years, increasing age had a small effect on the risk of all-cause mortality, whereas after the age of 50 years, increasing age significantly accelerated the increase in the risk of all-cause mortality. These findings not only enrich our understanding of the biological properties of MTC and their prognostic influences, but also provide an important basis for age-based personalised treatment strategies in clinical practice. In particular, for MTC patients over 50 years of age, the results of this study suggest that the risk of all-cause mortality is significantly increased, and therefore closer follow-up and monitoring in clinical management is recommended to detect and manage potential recurrence or metastatic risk in time to improve the long-term prognosis of patients.

## Data Availability

The original contributions presented in the study are included in the article/supplementary material. Further inquiries can be directed to the corresponding authors.

## References

[1] Liu D Wang W Wu Y Qiu Y Zhang L. LINC00887 acts as an enhancer RNA to promote medullary thyroid carcinoma progression by binding with FOXQ1. Curr Cancer Drug Targets. (2024) 24:519–33. doi: 10.2174/0115680096258716231026063704 38804344

[2] Watkinson JC British Thyroid Association. The British Thyroid Association guidelines for the management of thyroid cancer in adults. Nucl Med Commun. (2004) 25:897–900. doi: 10.1097/00006231-200409000-00006 15319594

[3] Thomas CM Asa SL Ezzat S Sawka AM Goldstein D. Diagnosis and pathologic characteristics of medullary thyroid carcinoma-review of current guidelines. Curr Oncol. (2019) 26:338–44. doi: 10.3747/co.26.5539 PMC6821118 31708652

[4] Wells SAJ Asa SL Dralle H Elisei R Evans DB Gagel RF. Revised American Thyroid Association guidelines for the management of medullary thyroid carcinoma. Thyroid. (2015) 25:567–610. doi: 10.1089/thy.2014.0335 25810047 PMC4490627

[5] Sahakian N Castinetti F Romanet P. Molecular basis and natural history of medullary thyroid cancer: it is (Almost) all in the RET. Cancers (Basel). (2023) 15. doi: 10.3390/cancers15194865 PMC10572078 37835559

[6] Xian KY Liu J. Progress and controversy on the extent of surgery for medullary thyroid carcinoma based on calcitonin levels. Zhonghua yi xue za zhi. (2024) 104:1755–8. doi: 10.3760/cma.j.cn112137-20231110-01059 38782745

[7] Mathiesen JS Kroustrup JP Vestergaard P Stochholm K Poulsen PL Rasmussen ÅK. Incidence and prevalence of sporadic and hereditary MTC in Denmark 1960-2014: a nationwide study. Endocr Connect. (2018) 7:829–39. doi: 10.1530/EC-18-0157 PMC6000757 29760189

[8] Jahantab MB Rastegar B Aria A. A case report of multiple endocrine neoplasia type 2B. Ann Med Surg (2012). (2024) 86:3016–9. doi: 10.1097/MS9.0000000000001867 PMC11060199 38694328

[9] Zhang H-F Huang SL Wang WL Zhou YQ Jiang J Dai ZJ. C634Y mutation in RET-induced multiple endocrine neoplasia type 2A: A case report. World J Clin cases. (2024) 12:2627–35. doi: 10.12998/wjcc.v12.i15.2627 PMC11135442 38817239

[10] Gild ML Clifton-Bligh RJ Wirth LJ Robinson BG. Medullary thyroid cancer: updates and challenges. Endocr Rev. (2023) 44:934–46. doi: 10.1210/endrev/bnad013 PMC10656709 37204852

[11] Roman S Lin R Sosa JA. Prognosis of medullary thyroid carcinoma: demographic, clinical, and pathologic predictors of survival in 1252 cases. Cancer. (2006) 107:2134–42. doi: 10.1002/cncr.v107:9 17019736

[12] Kondo T Ezzat S Asa SL. Pathogenetic mechanisms in thyroid follicular-cell neoplasia. Nat Rev Cancer. (2006) 6:292–306. doi: 10.1038/nrc1836 16557281

[13] Rahmani Samani M Zarif-Yeganeh M Mehrabi A Emami Razavi AN Sheikholeslami S Hedayati M. Expression of miR-127, miR-154, and miR-183 in Medullary Thyroid Carcinoma Tumors. Iran J Public Health. (2021) 50:391–6. doi: 10.18502/ijph.v50i2.5358 PMC7956075 33748004

[14] Roman S Mehta P Sosa JA. Medullary thyroid cancer: early detection and novel treatments. Curr Opin Oncol. (2009) 21:5–10. doi: 10.1097/CCO.0b013e32831ba0b3 19125012

[15] Cirello V. Familial non-medullary thyroid carcinoma: clinico-pathological features, current knowledge and novelty regarding genetic risk factors. Minerva Endocrinol (Torino). (2020) 46:5–20. doi: 10.1210/endrev/bnad013 33045820

[16] Hankinson TC. Short-term mortality following surgical procedures for the diagnosis of pediatric brain tumors: outcome analysis in 5533 children from SEER, 2004-2011. J Neurosurg Pediatr. (2015) 17:289–97. doi: 10.23736/S2724-6507.20.03338-6 26588456

[17] Dudley RWR Dudley RW Torok MR Patibandla MR Dorris K Poonia S. Pediatric low-grade ganglioglioma: epidemiology, treatments, and outcome analysis on 348 children from the surveillance, epidemiology, and end results database. Neurosurg. (2015) 76:313–9. doi: 10.1227/NEU.0000000000000619 PMC4333003 25603107

[18] Dudley RWR Torok MR Gallegos DR Mulcahy-Levy JM Hoffman LM Liu AK. Pediatric choroid plexus tumors: epidemiology, treatments, and outcome analysis on 202 children from the SEER database. J Neurooncol. (2014) 121:201–7. doi: 10.1007/s11060-014-1628-6 25297498

[19] Cahill KS Claus EB. Treatment and survival of patients with nonmalignant intracranial meningioma: results from the Surveillance, Epidemiology, and End Results Program of the National Cancer Institute. Clin article J Neurosurg. (2011) 115:259–67. doi: 10.3171/2011.3.JNS101748 PMC3256743 21529132

[20] Milano AF. 20-year comparative survival and mortality of cancer of the stomach by age, sex, race, stage, grade, cohort entry time-period, disease duration & Selected ICD-O-3 oncologic phenotypes: A systematic review of 157,258 cases for diagnosis years 1973-2014: (SEER*Stat 8.3.4). J Insur Med. (2019) 48:5–23. doi: 10.17849/insm-48-1-1-19.1 31609640

[21] Yu JB Gross CP Wilson LD Smith BD. NCI SEER public-use data: applications and limitations in oncology research. Oncol (Williston Park). (2009) 23:288–95. doi: 10.17849/insm-48-1-1-19.1 19418830

[22] Zhao Z Yin XD Zhang XH Li ZW Wang DW. Comparison of pediatric and adult medullary thyroid carcinoma based on SEER program. Sci Rep. (2020) 10:13310. doi: 10.1038/s41598-020-70439-7 32764626 PMC7413344

[23] Han S Georgiev P Ringel AE Sharpe AH Haigis MC. Age-associated remodeling of T cell immunity and metabolism. Cell Metab. (2023) 35:36–55. doi: 10.1016/j.cmet.2022.11.005 36473467 PMC10799654

[24] Fane M Weeraratna AT. How the ageing microenvironment influences tumour progression. Nat Rev Cancer. (2020) 20:89–106. doi: 10.1038/s41568-019-0222-9 31836838 PMC7377404

